# A simple, high throughput method to locate single copy sequences from Bacterial Artificial Chromosome (BAC) libraries using High Resolution Melt analysis

**DOI:** 10.1186/1471-2164-11-301

**Published:** 2010-05-12

**Authors:** Giang TH Vu, Peter DS Caligari, Mike J Wilkinson

**Affiliations:** 1 Institute of Biological, Environmental and Rural Sciences, Aberystwyth University, Penglais, Aberystwyth, Ceredigion, SY23 3DA, UK; 2 Sumatra Bioscience Pte Ltd, 8 Eu Tong Sen Street, #16-94/95 The Central, 059818, Singapore; 3 BioHybrids International Ltd, 11 Penrose Avenue, Woodley, Berkshire RG5 3PA, UK

## Abstract

**Background:**

The high-throughput anchoring of genetic markers into contigs is required for many ongoing physical mapping projects. Multidimentional BAC pooling strategies for PCR-based screening of large insert libraries is a widely used alternative to high density filter hybridisation of bacterial colonies. To date, concerns over reliability have led most if not all groups engaged in high throughput physical mapping projects to favour BAC DNA isolation prior to amplification by conventional PCR.

**Results:**

Here, we report the first combined use of Multiplex Tandem PCR (MT-PCR) and High Resolution Melt (HRM) analysis on bacterial stocks of BAC library superpools as a means of rapidly anchoring markers to BAC colonies and thereby to integrate genetic and physical maps. We exemplify the approach using a BAC library of the model plant *Arabidopsis thaliana*. Super pools of twenty five 384-well plates and two-dimension matrix pools of the BAC library were prepared for marker screening. The entire procedure only requires around 3 h to anchor one marker.

**Conclusions:**

A pre-amplification step during MT-PCR allows high multiplexing and increases the sensitivity and reliability of subsequent HRM discrimination. This simple gel-free protocol is more reliable, faster and far less costly than conventional PCR screening. The option to screen in parallel 3 genetic markers in one MT-PCR-HRM reaction using templates from directly pooled bacterial stocks of BAC-containing bacteria further reduces time for anchoring markers in physical maps of species with large genomes.

## Background

Whole genome sequence data is currently unavailable for the overwhelming majority of plant species, including most crops. For such cases, the integration of linkage and physical maps provides a vital information platform to accelerate processes such as positional cloning, comparative genome analysis, and clone-by-clone sequencing [1-3]. One key limiting step in compiling a comprehensive link between physical and linkage maps is the ability to locate large insert clones (e.g. from Bacterial Artificial Chromosome [BAC] or Yeast Artificial Chromosome [YAC] libraries) that contain the polymorphic markers used in linkage mapping. Traditionally, this has been achieved by colony hybridisation using (usually) radioactively-labelled cloned DNA, PCR products, or oligonucleotides [4,5]. There are several disadvantages of using colony hybridisation as a means of identifying clones that contain target DNA; these largely centre on the difficulty in setting appropriate hybridization conditions to minimise false positive and false negative results, but also include the need for appropriate facilities and procedures to handle radio-labelled probes [6]. These problems can be overcome if a PCR-based screening strategy is adopted using Sequence Tagged Site (STS) markers [6,7].

Several authors have argued that the efficiency of PCR-based screening can be greatly improved through the use of structured multidimentional BAC pooling strategies [7,8]. Compared with hybridization to high-density colony filters, the PCR screening of multidimentional BAC pools is less prone to the confounding effects of repetitive elements and tends to be more cumbersome when radiolabels are used [7]. Issues of error arise from both approaches, however, with the relative abundance of type 1 and type 2 errors varying according to post-hybridization wash stringency or PCR annealing temperatures respectively. Importantly, the number of independent amplifications required to determine a clone's address can be reduced by smart pooling strategies. The use of conventional PCR for BAC pool screening to anchor genetic markers to physical maps with a high throughput retains some shortcomings, most notably the common need to: i) isolate and normalise DNA from a large number of clones (the whole library) to ensure that sufficient template is available from each BAC for reliable amplification of single copy targets by conventional PCR. ii) anchor and score each marker separately (multiplexing is generally difficult). iii) use agarose gel electrophoresis as crude verification (by size) of the fidelity of target amplification. This last step is simple but comparatively slow and difficult to automate.

**Figure 1:**
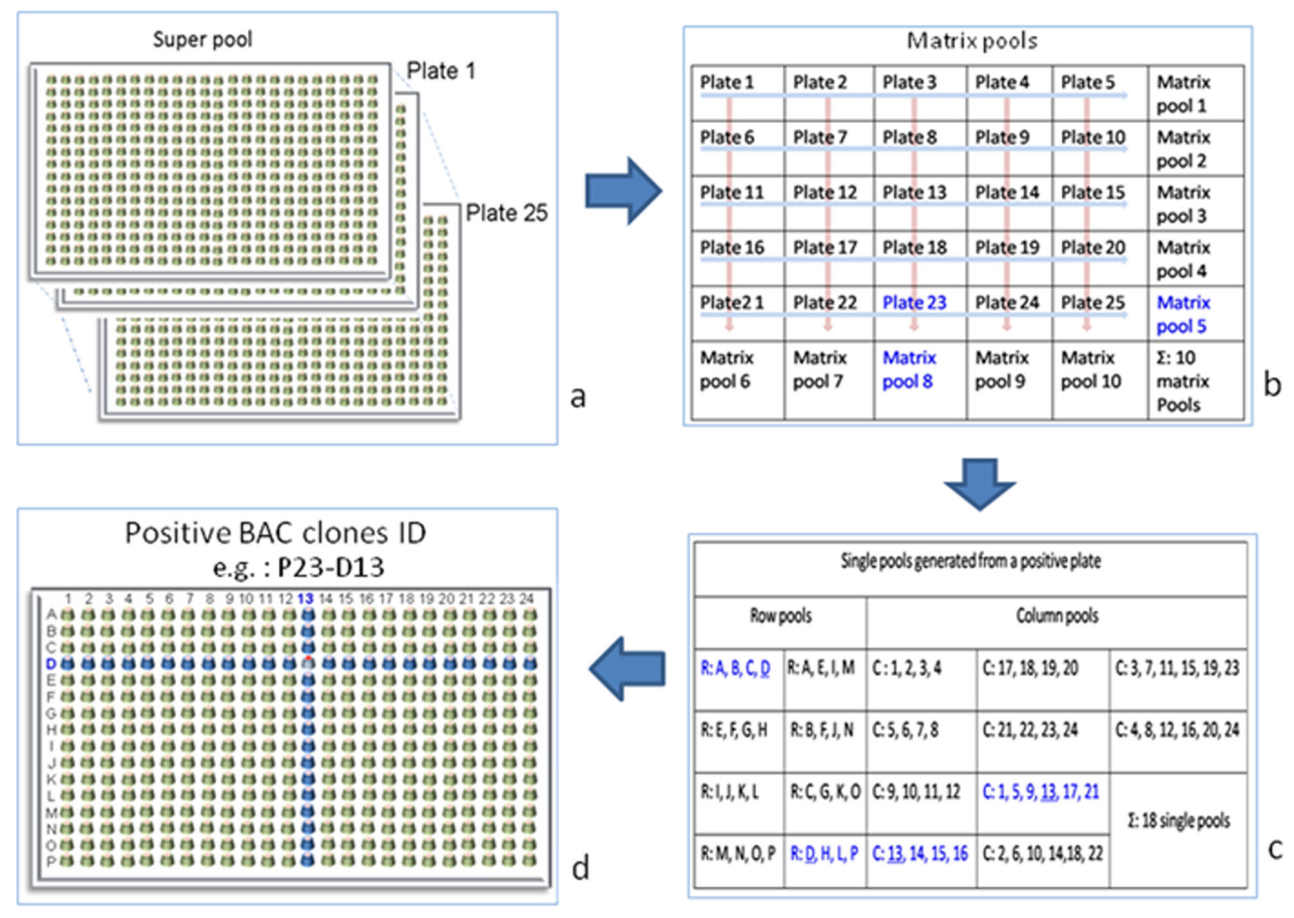
**Two-dimension BAC library pooling strategy**. (a) A super pool that combines twenty five 384-well plates. (b) Two-dimension matrix pools (horizontal and vertical ones) are created from each positive super pool. The MT-PCR-HRM screen of 10 pools for each matrix will identify the positive plate (e.g. plate 23) within 2 matrix pools. (c) Then, two-dimension single pools (column pools and row pools) are created from each positive plate. The row and column pools are arranged in a way that positive BACs will appear in two row pools as well as in two column pools. Thus, from a 384 well plate containing 16 rows (A to P) and 24 columns (1 to 24), at least 8 row pools (4 different rows each) and 10 column pools (6 of 4 columns and 4 of 6 columns each) have to be generated. The PCR-HRM screen of 18 single pools (8 row pools and 10 column pools) for each positive plate will identify the positive BAC clone (e.g. row D, column 13) (d) for the marker in question.

To overcome these limitations we describe a robust method for anchoring genetic markers in physical maps based on screening two-dimensional BAC pools (Figure 1) using a combination of a modified Multiplex-Tandem PCR (MT-PCR) and High Resolution Melt analysis (HRM). We demonstrate for *A. thaliana *that this strategy opens the possibility of direct screening of a BAC library using pooled freezing stocks for PCR with high throughput, at low cost, and a minimum risk of false positive/negative results.

## Results and discussion

### Rationale of the method

**Figure 2:**
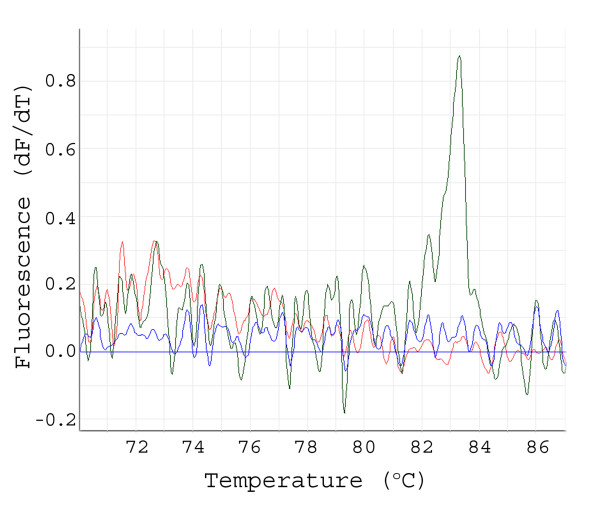
**The need for pre-amplification: MT-PCR-HRM using freezing stocks of a super pool (25 × 384 well plates)**. A first order differential plot showing failed amplification by direct PCR (35 cycles, red line) and in the negative water control (blue line) but a strong signal after preamplification applying MT-PCR-HRM (green line) for STS marker M45.

BAC library screening usually requires DNA extraction from the BAC pools [7,9] to increase template accessibility sufficiently to allow reliable amplification by conventional PCR. We addressed the problem of low DNA template in BAC pools using a modification of Multiplex-Tandem PCR (MT-PCR) (Figure 2). The MT-PCR protocol was originally developed to amplify rare transcripts from cDNA pools [10] but was subsequently adapted for use on genomic DNA samples [11]. The technique first comprises a truncated pre-amplification step that typically consists of 15-20 cycles so that PCR amplification is arrested in the log-linear phase and template concentration ratios are broadly retained. Importantly, this stage is highly amenable for multiplexing (up to 50 loci). After dilution, selective amplification is performed by conventional PCR (usually 30-35 cycles) for each locus separately using either identical primers to those used for pre-amplification (as here) or new primers targeting internal sites [11]. The value of MT-PCR lies in its ability to reliably amplify from low copy template within mixed samples. This property makes the technique highly attractive for BAC library screening for low copy targets and opens the possibility of direct screening from freezing stocks rather than from isolated BAC DNA.

**Figure 3:**
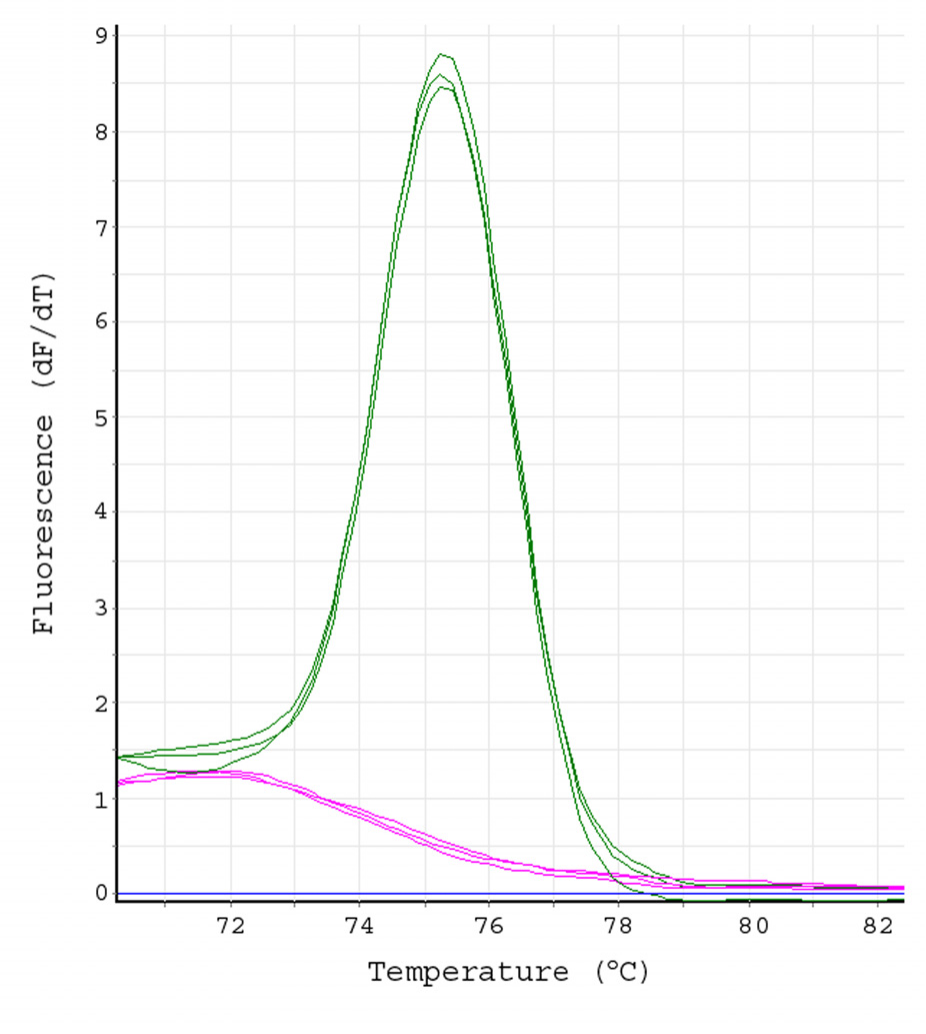
**Screen of Matrix pools by MT-PCR-HRM**. First order differential plots of a High Resolution Melt derived from a MT-PCR-HRM experiment for STS marker M106 (three replicates per treatment) performed on BAC matrix pools (5 × 384 well plates) containing a single target BAC (green line) or on the same matrix pool but lacking the target (pink line). All HRM analyses performed on a Qiagen Rotor-Gene 6000.

**Figure 4:**
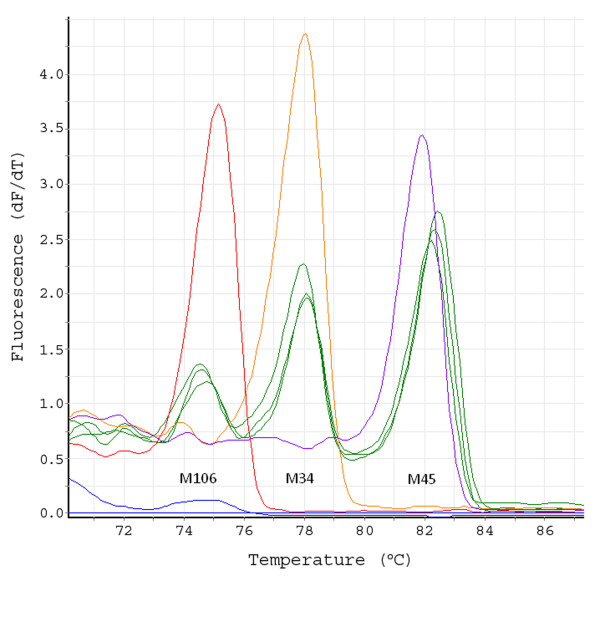
**Multiplexing 3 genetic markers in one MT-PCR-HRM screening reaction**. First order differential plots of a High Resolution Melt derived from a multiplex MT-PCR-HRM experiment (three replicates per treatment, green lines) performed on BAC matrix pools (5 × 384 well plates) using three independent loci as revealed on a Qiagen Rotor-Gene 6000. Positive control samples were generated using individual BAC clones containing each of the three loci (M106, red line; M34, orange line and M45, purple line). Negative control (blue line) lacked template DNA. Note that the positive control traces each occupy a diagnostic position on the trace and that the three experimental traces yield peaks corresponding to each of these positions (green).

There remains, however, a need for a high-speed, low-cost system to verify identity of the amplicons generated by PCR (i.e. remove type 1 errors through mis-amplification) and also to distinguish PCR products from primer dimers in a gel-free environment (also creating type 1 errors). High Resolution Melt (HRM) analysis is a powerful tool for high throughput mutation detection [12] and genotyping [13]. It therefore provides an attractive means of verifying the identity of PCR amplicons from BAC pools (Figure 3). The advantage of HRM over other genotyping technologies for this purpose reside in its simplicity, high speed and low cost, the capacity to detect differences based on size and sequence, and the consistency of data output over a wide range of targets [14,15]. HRM analysis monitors alteration in fluorescence caused by the release of an intercalating dye from the double-stranded DNA of PCR products as they dissociate with increasing temperatures. Differences between PCR products are revealed by melting temperature shifts representing minor sequence or size variations [14]. Recent advance in fluorescence-detecting instruments (e.g. the Qiagen Rotor-Gene 6000) allows HRM to be performed immediately following amplification and to be completed within 15 min. Careful selection of amplicons that have non-overlapping melt profiles also opens up the possibility of multiplexing HRM analysis (Figure 4). Multiplex pre-amplification and HRM analysis make BAC library screening easy and minimize the risk of false positive or false negative results that can appear when pooled freezing stocks are directly used for PCR.

Our MT-PCR-HRM-based protocol describes a rapid and robust way to identify low abundance positive clones within large insert libraries. A positive superpool is identified by N/25 MT-PCR-HRM reactions where N is the number of 384-well plates of the BAC library and a super pool contained 25 384-well plates (Table 1). A positive plate will be defined by two positive matrix pools after 10 MT-PCR-HRM reactions. The positive clone of a given plate is identified by the same 2D pooling strategy from four positive single pools (2 row and 2 column pools, Figure 1c) after 18 PCR-HRM reactions (Figure 1 and Table 2). In total N/25 + 28 MT-PCR-HRM reactions are needed for each marker when tested separately (Table 1). The number of MT-PCR-HRM reactions can be reduced when up to three suitable markers are combined into a single MT-PCR-HRM.

**Table 1:** Comparison of screening BAC library by conventional PCR and by MT-PCR-HRM.

Compared features	Screening by conventional PCR using 3D pooling strategy	Screening by MT-PCR-HRM using 2D pooling strategy
Maximum number of 384-well plates to be pooled in one super pool	10 384-well plates	25 384-well plates

Number of super pools to be tested to identify a positive superpool (N: The total plate number of the BAC library)	N/10	N/25

PCR reactions (PCR/PCR-HRM) needed to identify a positive super pool	N/10	N/25

Reactions needed to identify the plate ID for one positive super pool	10	10

Reactions needed to identify the clone ID from one positive plate	40	18

Total number of reactions needed to get positive BAC clone ID from whole library	N/10 + 50	N/25 + 28

Multiplexing possibility	No	Yes

Checking on agarose gel	Needed	NO

Cost	0.16 £/1 PCR reaction + cost for agarose gel electrophoresis	0.17 £/1 PCR-HRM reaction

Procedure duration to anchor 1 marker	12 h	3 h

Scoring data	Manually from gel photos	Semi-Automatic (figures and summary tables can be exported from HRM rotor system)

**Table 2:** MT-PCR-HRM pooling strategy to accommodate for variable genome size.

Organism	1C genome (Mb)	No 384 well plates	No super pools	No matrix pools	No single pools	Total required simplex MT-PCR-HRM	Total required triplex MT-PCR-HRM
*Brachypodium distachyon*	300	24	1	10	54*	64	22

Potato	850	66	3	30	54	87	29

Barley	5500	430	17	30	54	101	35

Wheat	16000	1250	50	30	54	134	45

Swine	2600	203	8	30	54	92	31

Cattle	3100	242	10	30	54	94	32

Estimated number of pools and MT-PCR-HRM reactions required to find a single marker (or 3 markers simultaneously) within the denoted genomes, assuming a 3× coverage and 100 Kb average insert size for the corresponding BAC libraries. Note: *1× coverage = 18 single pools (see Figure 1); 3× coverage = 54 single pools

### The practical example

We use *A. thaliana *as an exemplar to demonstrate the usefulness of MT-PCR-HRM in combination with a multidimensional BAC pooling strategy for the anchoring of markers into physical maps.

The experimental design comprises of three main steps: 1) pooling of BAC freezing stocks; 2) multiplex pre-amplification of BAC pools with up to 50 primer pairs; 3) localisation of positive BAC clones by identifying the positive subset of pools *via *selective amplification followed by HRM analysis (for each marker separately, or for up to three markers simultaneously) to confirm locus identity.

#### 1) Pooling strategy for PCR screening of a genomic BAC library

The size of large insert libraries is normally adjusted according to the haploid genome size, the mean insert size and the expected genome coverage of the library. A typical BAC library has approximately 100 Kb average insert size. It is recommended to use a BAC library with at least 3 times coverage for PCR-based screening purposes [5]. Such genomic BAC libraries are typically stored in microtiter plates in a 384-well format.

Several pooling strategies can be applied for PCR-based screening. The number of dimensions (D) deployed in a pooling strategy defines the number subsets of pools that may contain a specific BAC clone. Screening techniques based on conventional PCR normally require 3-D or 6-D pooling systems [5,7-10] in order to ensure reliable amplification from a single BAC clone. With the more sensitive and accurate MT-PCR-HRM technique, a two-dimension pooling strategy (Figure 1) is sufficient. For studies aiming to anchor genetic markers into physical maps of large genomes, the minimum number of screening reactions is decisive in setting labour and consumables costs. The pooling strategy proposed here allows screening of millions of clones with as few as the number of superpools plus 28 MT-PCR-HRM reactions (Figure 1). Based on our experience, each super pool should not comprise more than twenty five 384-well plates. Using 5 μL per BAC clone, a super pool contains in total 5 × 25 × 384 μL (= 48 mL). In our illustrative example, an *A. thaliana *library [16] was used that contained twenty four 384-well plates. To evaluate whether the enhanced sensitivity of this method has utility for organisms with much larger genomes, we simulated 'rare single locus' hits in a substantially larger library by creating a superpool containing 5 μL of a single positive BAC clone and 3200 ([25 × 384]/3) negative BAC clones of 15 μL each. We were able to exploit this approach to estimate the capability of the technique to detect single locus hits for genomes of much larger size (Table 2).

According to the proposed pooling strategy, the required number of super pools for a typical 3× coverage BAC library with a 100 Kb average insert size is estimated for several important genomes (Table 2). For genomes larger than 1500 Mb, the number of PCRs required to anchor one genetic marker is approximately equivalent. The number of plates per superpool can be varied in a certain frame (i.e., less than 25) according to the genome size of the organism under study.

#### 2) MT-PCR pre-amplification

Applying a suitable pooling strategy, the number of PCR amplifications required to identify the positive BAC clones can be reduced significantly. However, because of a low template concentration for STS sites and further dilution by *E. coli *DNA, conventional PCR-based screening may over-dilute single locus targets to such an extent that amplifications often fail. DNA extraction from the BACs is required for better template accessibility [7,9]. Instead, we introduced a multiplex pre-amplification step on super BAC pools (each containing twenty five 384-well plates) directly from freezing stocks, applying up to 50 primer pairs simultaneously. Before pre-amplification, it has to be confirmed that each primer pair applied works individually on total genomic DNA by PCR-HRM.

The pre-amplification increases the sensitivity and specificity of MT-PCR-HRM on pooled templates (Figure 2). Without pre-amplification a higher cycle number is generally required for conventional PCR (40 to >45 cycles), yielding an excessively high background to product ratio (Figure 2).

#### 3) Identification of marker-containing BAC clones by high resolution melting (HRM) analysis

HRM curve analysis after PCR-HRM provides a rapid, sensitive means for the BAC screening. The Rotor-Gene™ 6000 (Qiagen) with a real-time rotary analyzer prevents unwanted temperature deviation inside the thermal cycler [15,16]. Furthermore, intercalating dye improves HRM sensitivity, allows omission of the gel electrophoresis step and enhances true positive hit rates [16]. The positive BAC pools yield clear melting curves instead of a background line as obtained in the case of negative BAC pools (Figure 3). Because melting curve analysis can differentiate between divergent sequences, the option of a multiplex PCR for up to 3 loci is applicable to reduce consumables and labour (Figure 4). The entire MT-PCR-HRM run for a single locus (or for three loci together) can be completed within 90 minutes.

The same approach is applicable to organisms of much larger genome sizes (see Table 2). For example, using the tri-plex MT-PCR-HRM option in which three loci exhibiting non-overlapping melt profiles are combined into a single reaction, only 45 PCRs are required to screen approximately 480,000 clones of the 16 Gbp wheat genome for each of three markers represented in (an) individual BAC(s) (Table 2). Thus, this strategy potentially opens the possibility for high throughput-low cost screening across even very large genomes. Besides the integration of genetic and physical maps, the potential of this protocol is to support ongoing genome sequencing projects e.g., for barley http://barleygenome.org/, wheat http://www.wheatgenome.org/, potato http://www.potatogenome.net/, swine http://piggenome.org/, cattle http://www.bovinegenome.org etc.

## Conclusions

Here, the new MT-PCR-HRM technology has been applied to BAC matrix pools for simple, low-cost and high-throughput anchoring of genetic markers to physical maps. Using a BAC library of the model plant *Arabidopsis thaliana*, we were able to show that MT-PCR-HRM can get reliable amplification of single copy targets from freezing stocks equivalent to twenty five 384-well plate super pools. A two-dimension pooling strategy can locate a target clone within a superpool by 28 reactions. The method also allows effective multiplexing to screen, in parallel, 3 genetic markers in one MT-PCR-HRM reaction and is particularly suited for genome characterisation initiatives.

## Methods

### Primer design

Primer pairs for each genetic marker (Table 3) were designed using the Primer3 program http://www/genome.wi.mit.edu/genome_software/other/primer3.html. The optimal features of oligo primers are: 19 - 21 nucleotides in length, ~50% GC content, average melting temperature of 57-60°C and two C/G bases at the 3'end and one C/G base at the 5'-end.

**Table 3:** Primer sequences used to amplify *A. thaliana *genetic markers.

Marker ID	Forward	Reverse
M34	CCAACATCGTGGGTGTTGG	AAGGAGTCATGTTTGTAGC

M45	ATCGATCCCTACACTTCCC	ATTGGAGTGGCTTTATGCG

M106	GGAGATGTATGTGAGTGGG	AAGCATCTTAATGAAACCC

### Preparation of BAC pools from freezing stock of genomic BAC library

We used the *Arabidopsis *BAC library Mi/P1[17] in this protocol as an exemplar. The library plates stored at -80°C were thawed for 30 minutes and handled carefully in a flow cabinet to avoid contamination with clones from adjoining wells of the microtiter plate or exogenous contamination. After careful spin down of the plates, ethanol-wetted (70%) paper is used to clean the lip and walls of plates. Multichannel pipettes or robotic pipetting systems are used to take 5 μL of each colony for pooling into single, matrix or super pools.

### MT-PCR pre-amplification

Pre-amplification increases the concentration of the specific DNA templates in the freezing stock pools and therefore improves screening efficiency in the next step by avoiding false negative or false positive results. Modified MT-PCR is performed in 20 μL containing 10 μL of the supplied 2× Biomix (Biomix kit, Bioline), 2 μL freezing stock from the super pool, 5 μM of each primer (forward and reverse) multiplexed up to 50 markers and 4 mM MgCl_2_. Cycling conditions are 95°C for 10 min; then 20 cycles of 94°C for 20 s, 55°C for 30 s and 72°C for 1 min. The products are diluted in HPLC grade water to a final volume of 100 μL and stored at -20°C. To demonstrate the high multiplexing capacity of pre-amplification directly from pooled freezing stocks, 47 primer pairs (Additional file 1) from the list of 107 markers mapped to the 1.9 Mb FCA region of *A. thaliana *[18] were combined with three primer pairs shown Table 3 in one pre-amplification reaction. Subsequent application of the latter 3 primer pairs for selective MT-PCR-HRM screening of the BAC library identified the BACs harbouring the 3 markers.

### Library screening

To identify super pools (Figure 1a) that contain the positive BAC with the corresponding marker, the MT-PCR-HRM reactions (10 μL) for a single primer pair in question is performed in 0.1 mL tubes containing 2 μL of the diluted pre-amplification product, 5 μL of SensiMix*Plus *SYBR (Quantace) and 5 μM of each forward and reverse primer. The '3-step PCR with melt' should be setup in Qiagen Rotor-Gene 6000 (Qiagen) at following conditions: 95°C for 10 min, followed by 35 cycles at 95°C for 20 s, 57°C for 30 s and 72°C for 50 s. To gain fluorescence for each cycling at 72°C requires the operator to choose the 'green' option. High resolution melting analysis is performed at ramp from 65°C to 90°C, raising by 0.3°C each step, pausing 90 s at pre-melt condition as a first step and pausing 2 s for each step thereafter. To acquire melting fluorescence, 'green' needs be chosen. *In silico *computer graphics reveals the live PCR run, the concentration of PCR product at the stationary stage and the subsequent melt curves. First order differential plots of the melt curves of the PCR product are created by the software provided with the Rotor-Gene™ 6000.

To identify an individual plate, MT-PCR-HRM screening is to be performed with freezing stocks of matrix pools as template (Figure 1b). It is possible to perform multiplex MT-PCR-HRM with up to three markers as long as distinguishable melting curves can be obtained (see Figure 4). MT-PCR-HRM components and conditions are as in the super pool step. After surveying positive plates for every marker, positive BAC clones' ID (Figure 1d) will be identified by PCR-HRM using 2-D single plate pooling (Figure 1c) that pools across rows or columns to identify positive BAC clones. Markers that yield positive signals in one plate can be used for multiplex PCR. PCR-HRM components and conditions are as in the super pool step.

With the list of positive BAC clones for the marker in question, the anchoring process is completed.

## Authors' contributions

GV conducted the experimental procedures. GV and MW jointly conceived the work and all authors co-wrote and approved the manuscript.

## Supplementary Material

Additional file 1 **Multiplexing primers**. 47 primer pairs mapped to the 1.9 Mb FCA region of A. thaliana [18] were used successfully for pre amplification to demonstrate the high multiplexing capacity. Click here for file
