# Supplementary material for: A simple, high throughput method to locate single copy sequences from Bacterial Artificial Chromosome (BAC) libraries using High Resolution Melt analysis
Source: BMC Genomics. 2010 May 12;11:301. doi: 10.1186/1471-2164-11-301 (PMC2881884; doi:10.1186/1471-2164-11-301)
Supplement: Additional file 1 — Multiplexing primers. 47 primer pairs mapped to the 1.9 Mb FCA region of A. thaliana [18] were used successfully for pre amplification to demonstrate the high multiplexing capacity. [file 1471-2164-11-301-S1.DOC]

**Additional file 1**: **Multiplexing primers**

47 primer pairs mapped to the 1.9 Mb FCA region of A. thaliana [18] were used successfully for pre amplification to demonstrate the high multiplexing capacity.

| Marker | External forward primer | Reverse primer |
| --- | --- | --- |
| LR1 | TGGATGTCCAACGTCAGTGT | TGGTCCTCACAATCACCAGA |
| LR2 | GTCATGATCGCTGGAAAGGT | CCCTCAGCCAAGACAATGAT |
| AR1R | GCTGCAAGGTCTCCACCC | GTTCCAATGAAGAAGGTGCG |
| LR5 | TAAGGCGAAAGTGGAGGAGA | ACACAAACACAGGTGGTGGA |
| LR3 | TGGTTCATCACCAAAGTCCA | CTCCCTCGATGACCGATTTA |
| AR2R | CTGCTTACTCGTTCCTCC | CTGTTATGCACAGAACCACC |
| AR3R | GCATTGACAGTTCATCAGGG | GTCCATGTGGTTGTTGTGG |
| AR4R | CCAAATTGAAATCGAACCAGG | CTCAACTCTAACCATATTCGG |
| LR7 | TTGACATGGGTGATCCAGAA | TTGATCCTCCTCCTGTCCAC |
| LR8 | CGTTTGCTGCAGGTTTTACA | CCAGCGTACTTGGGAGAAAC |
| LR9 | TTCTCTGCCGATGGTCTTTT | AGCTCGCTGATTTCGGAGTA |
| LR10 | CAGTTGAATCCCGAAAAGGA | CTCCAGCCAGTAAAGCCTTG |
| LR11 | CGGGATTGAAGCTCCTTGTA | ATGCTTCTCCCCATTCCTCT |
| AR5R | CCTTCCTCGTGAGCAGCC | GCATGCCTTCTTCGTTGCC |
| AR6R | GCCAAAACACTCAACGTGG | GCTCTTGCAAGAACATTCGG |
| ARM1B | CCAAGACAGACTTCTACTCC | GGAGTAGTACTCGATCTTGG |
| ARM1A | GCTTCGAAATCTGGAGACC | CTTGCTTGTGAGTCTCTTCC |
| LR13 | CAACCTCTAGCGAGCCATTC | AGTGTGTGGAAGCTGCTGAA |
| LR12 | CGTTTCTTTGGGCTTGAGAA | TCCAACCTACACGATGACCA |
| AR7R | CGAAGAGTAAGCGAGGTGG | CAAGGAAGTGACTTAGGACG |
| AR8R | CAGAGATTTCACACTTATGGC | GTGGACGGACCTTAGTGG |
| LR15 | AACAAAGCCATCGATTTTGC | TGAGCCAACAAGTGGAAACA |
| LR14 | AACAAACCGCAGAGGTCATT | CAGACCCGCTTCTTGACTTC |
| ARM2B | GTCTCTTGAATCGTTGAGCC | GTTGCAGAAGAAGTACAAGC |
| ARM2A | GGTCTCAGAAGCTGAGCC | CAAGCTACTAGAGCAAAGGC |
| AR9R | GGGATTGGCTTCTATGTTGC | CTTCGTACTTACAAAGTAGG |
| LR16 | GCCGTCATCATCAAATCCTT | ACCGGTTTTGAACCTCTCCT |
| AR10R | GTGTCCGAGTTTGAATCCG | CTCGAGCCAATGATACTCG |
| AR11R | CATCCCACTAAACCGTTGG | GACCTGTTTCTCATGGTACC |
| ARM3A | GAAGCTATTGGCAATGCTGG | CCAGCAACTGCACAAGCC |
| AR20R | GGTTCAAGATTGGTAACATGC | CCTCTCAAAAGTTGATCTAGG |
| LR28 | TGGTTCTGGGACTGGAAGAG | GCCTCGCATGTTACCAATCT |
| ARM7A | GTGGAATGGATGACGACCC | CTTCGAGTTCAATCCCATCC |
| LR29 | CGTTCCACTTCAACCAACCT | AGCCGACTTTGCTGTGTTCT |
| ARM7D | GACCTCGAGGGATAGTTGC | GAGTCCCTCATGTTGGACC |
| ARM7C | CGAGATCCTATAGCAGTTGC | GTTCCCATCCTGGTTCTCC |
| AR21R | GAGGAAGTAGATGATCAAGG | CAGCTTCTGCATTGAATGCC |
| ARM8A | CAAGCAGGTCCACCACGC | GACGGAAGAAACCAAGACG |
| ARM8C | GACAGCTTCCAACATTTGGG | GCTGAACAATTGGCTGATCG |
| ARM8B | CTCAGATTCAGCTTTCTAGC | GCATCAATCACCATCTGTGC |
| ARM8D | GAAGGAAACAAGAGTCAAGG | GCTCTACTTGCTGTTAGTGG |
| LR31 | TTTGACTGATCAGGGAGACG | GCCGATGTAGATCCAATGCT |
| AR22R | GAAGCATTTGACTGATCAGG | CTTGCAGGTATAGCTTCTGC |
| LR32 | CTCTCGGCTACGACTTCACC | AACGATTGGTACGGATTTGC |
| LR33 | AATTGCTGGCCCTGATATTG | GCAGTCTATGCAGCAACCAA |
| LR34 | TCCCTAATCCTTCGTGGATG | TGTTGAAAGTGTTCCCAGCA |
| LR35 | TGCGAAAGCAGACAAAACAG | TCATCGAAGCCATACCAACA |
